# Supplementary material for: Development of a computer-aided design software for the quantitative evaluation of aesthetic damage
Source: PLoS One. 2019 Dec 18;14(12):e0226322. doi: 10.1371/journal.pone.0226322 (PMC6919621; doi:10.1371/journal.pone.0226322)
Supplement: S1 Code site — 1. (ZIP) [file pone.0226322.s001.zip › [EESC_jr][Projeto]Relatorio_07_21/vendors/select2/docs/_includes/options/core/amd-support.html]

## Can I use Select2 with my AMD or CommonJS loader?

Select2 should work with most AMD- or CommonJS-compliant module loaders, including RequireJS and almond. Select2 ships with a modified version of the UMD jQuery template that supports both CommonJS and AMD environments.

### How do I tell Select2 where to look for modules?

For most AMD and CommonJS setups, the location of the data files used by Select2 will be automatically determined and handled without you needing to do anything.

If you are using Select2 in a build environment where preexisting module names are changed during a build step, Select2 may not be able to find optional dependencies or language files. You can manually set the prefixes to use for these files using the `amdBase` and `amdLanugageBase` options.

{% highlight js linenos %}
$.fn.select2.defaults.set('amdBase', 'select2/');
$.fn.select2.defaults.set('amdLanguageBase', 'select2/i18n/');
{% endhighlight %}

### Select2 is being placed before jQuery in my JavaScript file

Due to a bug in older versions of the r.js build tool, Select2 was sometimes placed before jQuery in then compiled build file. Because of this, Select2 will trigger an error because it won't be able to find or load jQuery.

By upgrading to version 2.1.18 or higher of the r.js build tool, you will be able to fix the issue.

### Should I point to the files in `dist` or `src`?

Select2 internally uses AMD and the r.js build tool to build the files located in the `dist` folder. These are built using the files in the `src` folder, so *you can* just point your modules to the Select2 source and load in `jquery.select2` or `select2/core` when you want to use Select2. The files located in the `dist` folder are also AMD-compatible, so you can point to that file if you want to load in all of the default Select2 modules.
